# Respiratory system mechanics in one-lung ventilation using double-lumen tubes

**DOI:** 10.1186/s40635-022-00450-x

**Published:** 2022-06-02

**Authors:** Jakob Wittenstein, Marcelo Gama de Abreu, Robert Huhle

**Affiliations:** 1 Department of Anesthesiology and Intensive Care Medicine, University Hospital Carl Gustav Carus, Technische Universität Dresden, Dresden, Germany; 2 Department of Intensive Care and Resuscitation and Department of Outcomes Research, Anesthesiology Institute, Cleveland Clinic, Cleveland, OH USA

## In response to Enk et al. ICMx 9:26 https://doi.org/10.1186/s40635-021-00392-w

We thank Enk and colleagues for the interest in our study comparing respiratory mechanics and gas exchange during one-lung ventilation (OLV) with flow controlled ventilation (FCV) and volume controlled ventilation (VCV) [1]. We know that, during FCV, alveolar and tracheal pressures differ. However, this applies to VCV in our study as well, where the end-expiratory flow was not zero. This is supported by the fact, that we found an increased intrinsic positive end-expiratory pressure (PEEP) during OLV using VCV [2]. During FCV, the compliance of the respiratory system is determined by performing an inspiratory hold manoeuvre every 10th breathing cycle. Thereby, the tracheal plateau pressure can be measured quasi-statically, and values should be comparable to those measured during VCV. However, this is not taken into account in the correction of compliance calculation as proposed by Enk and colleagues [1].

In our study, compliance did differ significantly during FCV and VCV [2]. Yet, gas exchange did not differ significantly between FCV and VCV, whereas FCV yielded higher ventilation efficacy. We disagree with Enk and colleagues that higher ventilation efficacy indicates higher dynamic compliance. During mechanical ventilation, hypoxemia is caused by hypoventilation and low ventilation–perfusion matching (V/Q). In contrast, impaired CO_2_ elimination results from high V/Q [3]. During FCV, dead space ventilation is reduced due to the relatively small inner diameter of the Tritube, as compared to the inner diameter of the double-lumen tube (DLT). Furthermore, emptying of lung units with different time constants is improved due to lower expiratory flow. Moreover, expiratory alveolar pressure is kept for a longer time above the alveolar closing pressure at the lower constant expiratory flow.

Finally, we disagree with Enk et al. that improved compliance must be followed by improved gas exchange. The turbulent nature of airflow during VCV, which is common when using a DLT (see analysis below), CO_2_ removal can be less effective as compared to a non-turbulent flow profile, such as during FCV. In fact, another group reported better ventilation with FCV compared to VCV during two lung ventilation, without increased compliance of the respiratory system [4]. It is conceivable, thus, that even at lower lung volume and total PEEP, ventilation was more efficient with FCV than VCV.

## Resistance of the double-lumen tube

We agree with Enk et al. that the computation of DLT resistance during VCV has important limitations. Certainly, direct measurement of airway pressure at the proximal end of the DLT during VCV would have been ideal. This, however, would have increased the complexity of the experimental set-up and reduced effective internal DLTs cross-section available to flow during VCV.

**Fig. 1:**
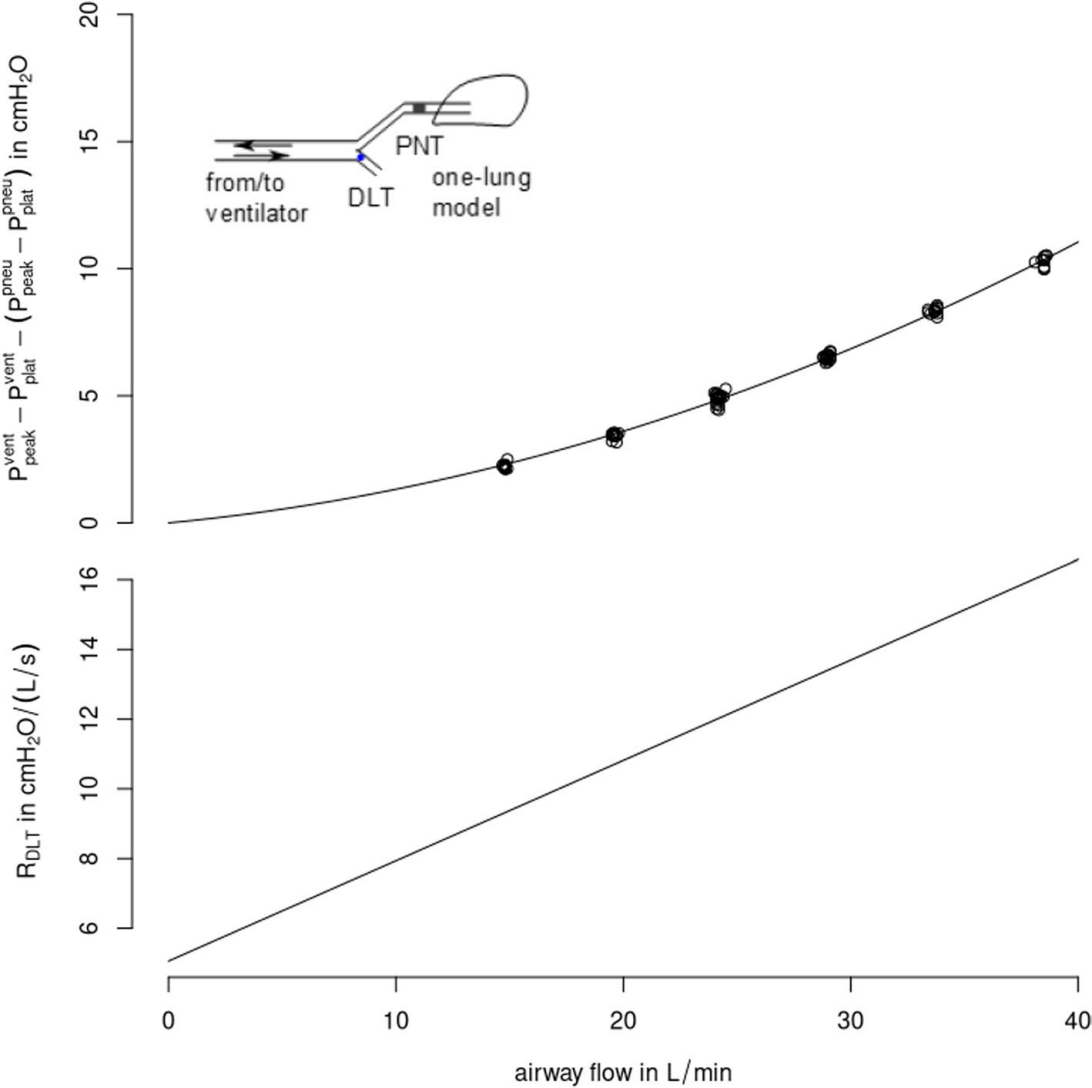
Measured and modelled pressure drop over double-lumen tube (DLT) resistance from ventilator (*P*_vent_) to tracheal (*P*_trach_) measured using a pneumotachograph (PNT) side of the DLT as well as corresponding DLT resistance (*R*_DLT_ lower row) over airway flow

Furthermore, we want to highlight that airflow was turbulent in our DLT. For the DLT used in our study (inner diameter 5.4 mm) and a flow of 25 l/min, a Reynolds number (Re) maybe derived theoretically from

$${\text{Re}}=\frac{\dot{V}\cdot \rho \cdot 2}{r\cdot \pi \cdot \eta }=6523,$$

with flow $\dot{V}$, the density of air *ρ* = 1.213 kg/m^3^, inner tube resistance *r* = 2.7 mm and dynamic viscosity of air *η* = 1.827·10^–5^ kg/(m·s). This value is higher than Re of 2000 (indicating the cut-off for laminar flow). Therefore, resistance in the DLT is flow-dependent, which was confirmed by bench tests performed on a test lung in a set-up chosen to match experimental settings as close as possible (VCV, tidal volume of 244 ml, I:E = 1:1, respiratory rate 15 min^−1^_,_ single lung test lung, compliance = 16 mL/cmH_2_O and resistance = 15 cmH_2_O·s/L, total lung volume 2 L, Adult Demonstration Lung Model, IngMar Medical, PA, USA, Fig. 1). The pressure decay over our DLT was higher than the one assumed by Enk et al. even at a flow rate of 24 L/min (3.6 vs. 2.5 cmH_2_O) (Fig. 1).

The corresponding Rohrer’s equation relating pressure drop over a tube (ΔP) to $\dot{V}$:

$$\Delta P={K}_{1}\cdot \dot{{V}^{2}}+{K}_{2}\cdot \dot{V}$$

with the two parameters K_1_ and K_2_ quantifying quadratic and linear influences of airway flow, respectively. *K*_1_ and *K*_2_ were parametrized using positive constant flow rates of 40, 35, 30, … 20, 15 L/min. Resistance of the DLT (R_DLT_) was determined accordingly by dividing Rohrer’s equation concerning airway flow:

$${R}_{DLT}\left(\dot{V}\right)=\frac{\Delta P}{\dot{V}}={K}_{1}\cdot \dot{V}+{K}_{2}.$$

According to the parametrized Rohrer’s equation, at an airway flow of 25 L/min, the DLTs resistance resulted in *R*_DLT_ = 12.3 cmH_2_O·s/L. Therefore, DLT resistance during VCV did account for ~ 50% of the total difference in airway resistance between both ventilation modes.

Most importantly, the linear theory of lumped parameter modelling of the respiratory system (electric circuit analogy) implies that consideration of additional resistance due to the DLT will not affect determined compliance. Nevertheless, due to non-linearity in the respiratory system, differences arose in compliance (DLT correction—no DLT correction) of − 0.73 ± 0.24 ml/cmH_2_O (*P* < 0.001 according to paired Wilcoxon test, data from all measurement points during VCV) indicating that compliance during VCV was actually minimally reduced using the approach described above, and thus favoured FCV with-in the comparison.

## Inertance of double-lumen tubes

Another important mechanical property of any tube, but especially DLT, next to their resistance is its inertance *I* that is proportional to the length *l* and anti-proportional to the inner radius *r* of the DLT:

$$I=\frac{\rho \cdot l}{\pi \cdot {r}^{2}}=0.24\frac{{cmH}_{2}O{s}^{2}}{L},$$

with the density of air *ρ*. If *I* is ignored during modelling respiratory mechanical parameters based on the respiratory signals as measured at the ventilator, it might potentially increase the error made during the determination of respiratory system compliance and resistance.

## Respiratory system mechanics and double-lumen tubes

Respiratory system compliance *C*, resistance *R* and *I* were determined on signals acquired at the ventilator and the pneumotachograph during the bench test, as described above, by non-linear least square optimization using Matlab (Mathworks Inc., Natick, MA, USA).

The modelling error expressed as the root-mean-square error (RMSE) was not affected by the model, but was significantly reduced for signals measured at the pneumotachograph. Determined resistance difference between ventilator and pneumotachograph acquired signals more closely resampled resistance of the DLT as determined by Rohrer's equation, above. Differences between *I* determined at the ventilator and the pneumotachograph agreed closely with the theoretical value of the DLTs *I* determined above. Finally, compliance (16 mL/cmH_2_O) was underestimated when determined based on signals measured at the ventilator independently of the model (Table 1).

**Table 1:** Respiratory system mechanic variables according to different compartment models

Model	Signals measured at	RMSE (cmH_2_O)	*C* (mL/cmH_2_O)	*R* (cmH_2_O·s/L)	*I* (cmH_2_O·s^2^/L)
RC	Ventilator	1.0 ± 0.5	19 ± 1	26 ± 6	n.a
	PNT	0.4 ± 0.2	17 ± 1	15 ± 4	n.a
RCI	Ventilator	0.9 ± 0.5	18 ± 1	25 ± 6	0.35 ± 0.1
	PNT	0.4 ± 0.2	16 ± 1	13 ± 4	0.10 ± 0.0

Values are mean ± standard deviation from 80 cycles during an inspiratory flow ramp down trial with flow values at 40, 35, 30,…, 20, 15 L/min, tidal volume of 244 ml, I:E = 1:1 and respiratory rate set to 15 min^−1^. Compliance (*C*), resistance (*R*) and inertance (*I*) as determined based on signals acquired at the ventilator and the pneumotachograph (PNT) using a two- (RC) and a three-compartmental model (RCI)

We provide new data from bench evaluation that elucidate respiratory mechanics during OLV with a DLT conforming theoretical derived values of inertance and resistance indicating overestimation of resistance as well as overestimation of compliance of the respiratory system. This should be considered when comparing different ventilation modes, measurement locations and devices.

Thus, in our study [2] compliance was overestimated during VCV, due to difference of location of airway flow and pressure measurement, favouring VCV in the comparison with FCV. However, this does not have any consequence on our conclusion that FCV showed higher ventilation efficacy with reduced mechanical power compared to VCV. In fact, without overestimation of compliance during VCV, the difference in mechanical power between both ventilation modes would have even been augmented.

## Data Availability

The data supporting this conclusion of this article are available from the authors upon reasonable request.

## References

[1] Enk D, Abram J, Spraider P, Barnes T (2021). Dynamic compliance in flow-controlled ventilation. Intensive Care Med Exp.

[2] Wittenstein J, Scharffenberg M, Ran X, Keller D, Michler P, Tauer S (2020). Comparative effects of flow vs. volume-controlled one-lung ventilation on gas exchange and respiratory system mechanics in pigs. Intensive Care Med Exp.

[3] Petersson J, Glenny RW (2014). Gas exchange and ventilation–perfusion relationships in the lung. Eur Respir J.

[4] Schmidt J, Wenzel C, Mahn M, Spassov S, Cristina Schmitz H, Borgmann S (2018). Improved lung recruitment and oxygenation during mandatory ventilation with a new expiratory ventilation assistance device: A controlled interventional trial in healthy pigs. Eur J Anaesthesiol.

